# Implementation Outcomes of National Convergence Action Policy to Accelerate Stunting Prevention and Reduction at the Local Level in Indonesia: A Qualitative Study

**DOI:** 10.3390/ijerph192013591

**Published:** 2022-10-20

**Authors:** Dewi Marhaeni Diah Herawati, Deni Kurniadi Sunjaya

**Affiliations:** Department of Public Health, Faculty of Medicine Universitas Padjadjaran, Bandung 40161, Indonesia

**Keywords:** implementation outcome, convergence action policy, stunting prevention and reduction, local level, Indonesia

## Abstract

The study aims to explore the implementation outcome variables of Indonesia’s national policy convergence action in the stunting reduction intervention at district, sub-district, and village levels. The study design was qualitative with an implementation research approach at District Cirebon, Indonesia. Data were collected through in-depth interviews, focus group discussions, study documents, and 6 months of participant observation. We recruited 172 respondents. The assessment instrument used was formed on was implementation outcomes variables. Data were analyzed through coding, categorizing and thematic content analysis based on a predetermined theme. Comparative cross district activity-site analysis was applied between sub-districts and villages. The implementation outcome variables for the convergence action policy were performed well at the district level, in line with the central government’s adequate regulation, control, and budget. Meanwhile, the sub-district and village levels only performed aspects of acceptability, appropriateness, and coverage for specific interventions. The acceptability level in the village was only partially running. The barriers at the sub-district and village levels were issues of commitment, staff capacity, and poor coordination. Superficial understanding and capacity weaknesses drove the convergence of the stunting reduction responsibility back into the burden of the health sector at the forefront. Local politics also colored the implementation in the village.

## 1. Introduction

Indonesia is currently the country in Southeast Asia with the highest estimated prevalence of stunting at 31.8%, followed by the Philippines (28.7%), Myanmar (25.2%), Malaysia (20.9%), and Thailand (12.7%) [1]. Stunting in early life results in reduced cognitive function, low productivity, obesity, and chronic disease [2,3]. Stunting is an indicator of children’s well-being and a marker of developmental and human resource inequalities [2,4,5]. 

Stunting seems to describe a downstream problem, which is the health sector’s responsibility, while the main problem accumulates upstream, where various sectors are responsible for it. The Government of Indonesia (GOI) has adopted the collaborative framework and multisectoral commitment of Scaling Up Nutrition to end malnutrition in all its forms, including stunting [6]. GOI has implemented various policies and programs for stunting reduction. One of the policies carried out nationally is the convergence action to accelerate the prevention and reduction in stunting, which involves the entire government, and private, academic, and community sectors from the central to the district/city level [7,8]. Practically, convergence actions are a strategy and effort to unite various programs from various sectors for one main goal, to focus on stunting prevention and reduction. 

The convergence action’s strategy and policy align with the ‘nutrition-specific’ (predominantly related to the health sector) and ‘nutrition-sensitive’ (related to non-health sectors) intervention program, which is carried out in an integrated and comprehensive manner by all stakeholders [7,8]. This integrated approach to priority targets in focus areas has succeeded in preventing and reducing stunting [9]. The converged multi-sector approach has been proven to be able to reduce the prevalence of stunting in Peru, Senegal, and Nepal through leadership, political commitments, and participation of all sectors, public and private [10,11,12]. Policies, programs, and interventions involving various sectors have made a major contribution in accelerating the reduction in stunting [13]. The prevalence of stunting in Indonesia has decreased from 37.8% (2013) to 27.6% (2019) and 24.4% (2021).

The convergence of nutrition-specific and -sensitive interventions can reduce the prevalence of stunting from 0.7–2% per year [14], and studies in Banggai District, Indonesia have shown that the interventions can reduce the prevalence of stunting by 2.18% per year [15]. Multisectoral interventions in sub-Saharan Africa can consistently improve household food security and food diversity [16]. Inter-sectoral convergence is a process of achieving efficiency, quality of coverage, and effectiveness to achieve goals through a holistic approach [17].

The GOI has set a target to reduce stunting prevalence from 24.4% in 2021 to 14% in 2024. One of the implementation activities is the performance appraisal of district governments (as the agents) by the GOI (as the principal) based on convergence actions. The District of Cirebon is one of the districts targeted as stunting focus areas by the GOI in 2019. A Cirebon District survey in 2019 showed a stunting prevalence of 25.06% [18].

The assessment of policy implementations and interventions is very important for feedback and tools for policy/program improvement. Implementation research can consider all aspects of implementation, including factors affecting implementation, implementation process, and implementation results, including potential solutions for the health system [19]. Research implementation is very effective in building the capacity of the involved stakeholders to achieve sustainable outcomes [20]. One of the tools is through an outcome implementation assessment, where the variables consist of acceptability, adoption, appropriateness, feasibility, fidelity, implementation cost, coverage, and sustainability [19].

The study aims to explore the implementation outcome variables of the national convergence policy in accelerating stunting prevention and reduction at the operational level. The research questions include aspects that occur in policy implementation, whether the strategy can be implemented at the operational level, and the policy actors who are responsible for convergence action in stunting prevention and reduction interventions in local and operational settings. The results of the research are intended for practitioners, government, and academics who work in a multi-sector approach to policies and programs, specifically stunting reduction, in achieving the Sustainable Development Goals (SDGs).

## 2. Materials and Methods

### 2.1. Research Design

The involvement of stakeholders including academics in the implementation is expected to help accelerate efforts in stunting reduction and prevention programs. In 2019, academics who were members of a unit called the Research Working Group (RWG) were assigned to assist local governments in implementing stunting reduction policies for nutrition-specific and -sensitive interventions in focus areas for 6 months. This project was called District Stunting Reduction Assistance (DSRA). The Ministry of Health financed all the facilitation activities of this study. 

RWG accompanies, observes, facilitates, participates, communicates, and gets feedback on every convergence action activity at the district, sub-district, and village levels. Activities that were run for 6 months were carried out together by the local government and RWG. Academic engagement was very intense and complementary to program strengthening. Academics helped conduct research for the benefit of strengthening strategies and policies at the district level.

In line with assistance and facilitative activities, we conducted a review of the policy implementation for the purpose of preparing policy recommendations as part of the umbrella study. The study design was qualitative with an implementation research approach in a district setting [19] and a pragmatism paradigm [21]. The study was conducted in District Cirebon, Indonesia, from June to November 2019. The researchers worked in a real situation where policy implementation was applied and occurred.

### 2.2. Study Context

The Cirebon district is located on the most populated island in Indonesia, in the west and north of the island of Java. There is a total population of 2,189,785 inhabitants and 10.06% of them are classified as poor based on the World Bank criteria. The number of children under five is 178,308, and 25.06% of them suffer from stunting. The district consists of 52 sub-districts and 412 villages.

Indonesia adheres to a decentralized political system and has 3 levels of government: national, provincial, and district/city. Each level has its autonomy, but provinces and districts in the regions must be in harmony with central policies. Under the district government, there are sub-districts and villages. A very interesting frame is that villages have their autonomy as well, while sub-districts are only district administrators, but coordinate district programs for villages in their area.

National policies are derived in the form of vertical programs that must be implemented by districts/cities. The national budget is allocated to the districts through the General Allocation Fund scheme. The district, as well as villages, can independently collect and manage funds regarding the national regulation.

The research focus on the implementation of convergence policies was to accelerate stunting reduction. It covered 8 convergence actions: (1) situation analysis, (2) activity plan, (3) consensus meeting, (4) the role of village decree, (5) human development cadre facilitation, (6) data management system, (7) measurement and publication, and (8) annual performance review.

### 2.3. Subject

All elements at the district level were subjected to assistance and observation, but for sub-districts and villages, this only applied to those that were designated as a locus of stunting. This location (district or village) was determined and designed by the government as an integrated target for the stunting reduction acceleration program. 

The sub-district level unit analysis was on 6 sub-districts that had stunting loci villages, while the village level unit analysis was on 10 stunting loci villages. Subjects in the district were all representatives of the Multi-Sector Agency. The other subjects were the Major of the district, the District Secretary, the Head of the Public Health Center (PHC), some non-government organizations, health cadres, and families with stunted toddlers. The number of research subjects at the district level was 49 participants, 58 participants at the sub-district level, and 65 participants at the village level. The total research subjects were 172 participants—this was the number of subjects recruited in the 6 months of research and facilitation. 

### 2.4. Data Collection

Data were collected in a predominantly qualitative form through in-depth interviews (IDI); serial focus group discussions (FGD); observations; and study documents in the district, 6 sub-districts, and 10 stunting loci villages. The IDI was carried out including the first-line district leaders: the Major and the District Secretary. It also involved the key subjects: parents with stunted toddlers and village chiefs. The activities that were carried out included 9 workshops/FGDs, 2 times at the sub-district level and 2 times at the village level.

An assessment was carried out twice at the beginning of the activity and at the end of the activity so that the baseline and end-line data were obtained. Data collection activities, both FGDs and document studies, began with situation analysis, data collection, and a budget review from all district officer agencies at the district, sub-districts, and village levels. 

Thus, the data we collected were in line with the district-assistance activities. For this study, we were especially focused on data collection related to the variables of the implementation outcome in each activity of implementing; in addition, we were focused on the convergence implementing outcomes in each activity of convergence action strategies until the lowest level, the family of stunting toddlers. 

### 2.5. Data Analysis

Qualitative data analysis was carried out through thematic content analysis [22]. Data from interviews, FGDs, and field notes were transcribed. Then, the analysis process was carried out through coding and categorization [23]. The resulting categories were matched and grouped into themes. We used computer-assisted data processing. The predetermined themes followed the implementation outcome variables, as mentioned in the introduction. Thick descriptions were presented on each theme.

In addition, mapping was also carried out for 8 convergence action activities at the district, sub-district, and village levels. A before-and-after comparative analysis was conducted at the district level (Table 1). Meanwhile, comparative cross-site analysis was carried out at the sub-district (Table 2) and village levels (Table 3) [24]. The focus of this research was more on the implementation of sub-national policies. However, during the course of the observation process, the issue of the role of the private sector in the primary care system for specific nutrition interventions prominently emerged (Table 4). Thus, a more in-depth analysis of this issue was carried out.

The triangulation of sources and methods was the main tool to support research credibility and confirmability [25]. The support of trustworthiness has been described in the research context (transferability aspects), as well as the subject description and data collection (dependability aspects).

## 3. Results

### 3.1. Acceptability

Early in the facilitation of the project by the RWG, many stakeholders at the district and sub-district levels did not properly understand the definition of stunting, the impact of stunting, nor the convergence action policy. District and sub-district levels had not formed the Stunting Reduction Acceleration Team (SRAT), as they should have been, according to regulations. This unit was one of the indicators of convergence actions. 

The local government accepted the stunting reduction policies and convergence actions from the central government as orders that must be obeyed and implemented, but they did not fully understand why they should pay more attention to ‘new’ policies. Stunting prevention and reduction programs and activities have been strengthening since 2010. However, since 2017, they have been changed to a stunting reduction convergence policy. 

The central government had, indeed, conveyed what they called the socialization of stunting reduction policies, but in fact, it was more of a dissemination of information only. To arrive at a proper implementation of national policy, it was firstly important to assess the extent to which ‘local agents’ in the district understood.

“I just understood after getting an explanation about the meaning of stunting. I don’t want the next generation not to have a good capacity. I want our toddlers to grow up healthy and intelligent to become a great future generation. And I ask the head of the District Development Planning Board (DDPB) that the budget for handling stunting next year will be increased, and immediately form the SRAT”.

(R2, district)

After understanding stunting, the impact, and convergence policies of accelerating stunting reduction, district and sub-district level stakeholders showed that they can follow the national policy well. However, some of them still have not changed their comprehension properly. After 6 months of assistance, the district and sub-district levels formed the SRAT, but not in all villages.

“As the coordinator of district development planning, we only understood after assistance from the University Team. We will soon form SRAT, currently, we have drafted the Decree”.

(R3, district)

“We admit that we actually don’t really understand the meaning of stunting, its impact, and the convergence policy for dealing with stunting. As local government officials, we accept this convergence policy, we have formed a sub-district level SRAT”.

(R56, sub-district)

The understanding of village-level stakeholders about stunting, its impact, and the convergence actions policy had not been very good, even though assistance had been carried out by the RWG for 6 months. At the end of the assistance, only 50% of villages formed teamwork.

“I haven’t formed SRAT yet, because I don’t know if creating teamwork can reduce stunting in my village”.

(R112, villages)

### 3.2. Adoption

All stakeholders at the district level at the end of the facilitation period carried out convergence actions for nutrition-specific and -sensitive interventions. This was moderated by the encouragement of DSRA. In addition, it was also reinforced by the central government, represented by the Ministry of Home Affairs and the provincial government through an annual assessment. District stunting program improvement became the performance of the district Major. However, at the sub-district and village level, convergence actions had not been effectuated correspondingly because there were many hindrances such as issues of understanding, commitment, and politics.

According to respondents, when nutrition-specific and -sensitive interventions at the sub-district level have not yet been implemented, it results in increasing the burden of the PHC. Sub-district stakeholders perceive that this work (stunting program) is the task of the PHC. Supposedly, nutrition-sensitive interventions are the duties of the education, environment, and food security sectors at the sub-district level. On the other hand, nutrition-specific interventions carried out by PHC have not involved private primary healthcare.

“We have carried out convergence actions, because of assistance and support from the university team which is amazing. The central and provincial governments conduct a convergence assessment and this becomes the district’s performance. We also plan to make local regulations to accelerate stunting reduction as well as local regulations on the use of village fund allocation that is used for nutrition specific and sensitive n intervention activities”.

(R4, district)

“We haven’t done 8 convergence actions, we will study first, we need assistance and guidance from the district government or district SRAT”.

(R67, sub-district)

“Politics at the village level is very high, when the previous village chief is not re-elected then all health activities will stop”.

(R143, villages)

“When the village chief is not in the same party as the major… then the village head is a bit lazy to run the major program”.

(R169, villages)

### 3.3. Appropriateness

All district-level, sub-districts, and villages stakeholders state that nutrition-specific and -sensitive interventions that are integrated are very appropriate in the handling and prevention of stunting. Stakeholders assert that the cause of stunting is due to a lack of food intake and the presence of infectious diseases. The latter is related to uninhabitable houses, a lack of clean water, open defecation-free (ODF) that remains low, unavailability of household food, etc. The whole cause of the intervention cannot be carried out by the health sector only, therefore, nutrition-sensitive interventions must be integrated with nutrition-specific interventions.

“We recognize that nutrition- specific and sensitive interventions integrated into the eight convergence actions are very appropriate in addressing stunting issues”.

(R11, district)

“Solving the problem of stunting cannot be solved by the health sector only, other sectors and the community must be involved”.

(R72, sub-district)

“Yes, convergence action is right to deal with stunting, there is a specific and sensitive integration”.

(R129, villages)

### 3.4. Feasibility

District, sub-district, and village level stakeholders said that this convergence policy was very feasible to do because, in fact, the policy is operationalized in activities that have been carried out regularly every year. However, all stakeholders realize that being integrated requires time and a process to lower their sectoral ego. So far, the handling of nutrition-specific and -sensitive interventions has not matched each other, such as the assistance in making toilets, healthy houses, and clean water constructions instead of at the locations of stunting toddlers or places where diarrhea or chronic infectious diseases occur.

“We realize that all this time we have been very sectoral, thinking less about the impact on society, hopefully in the future we will improve our intervention so that it can be more targeted”.

(R39, district)

“Yes, currently the construction of healthy houses is not in the place of toddler affected by stunting”.

(R74, sub-district)

“This stunting integration activity is not easy, we need time to explain it to all stakeholders”.

(R129, villages)

### 3.5. Fidelity

The district-level, after assistance, succeeded in carrying out the convergence action policy implementation represented by the nutrition-specific and -sensitive intervention planning in an integrated way. Planning made at the district level was coordinated by DDPB so that the stunting reduction plans made could be integrated. However, the majority of sub-district and villages have not yet implemented the convergence action policy because they have not been able to make integrated nutrition-specific and -sensitive intervention planning. The barrier comes from the lack of capacity of officers at the sub-district and village levels and they have never received integrated planning training.

“The sub-district has difficulty in planning, we don’t know how to make an integrated plan, and we haven’t received any training”.

(R52, sub-district)

“Villages have not been able to plan, we have never been trained in planning, we really need training”.

(R136, villages)

The operationalization of central government policies is based is in the village. They are the smallest government unit led by the village chief. The number of human resources and the level of education (staff) in the village government are generally limited. This weak capacity results in limited village-level stunting operational management. The role of the subdistrict in filling the capacity gap in their villages is one of the important strategies.

### 3.6. Implementation Cost

The implementation of stunting reduction as a national program requires a large budget. In general, the central government allocates the national budget through the General Allocation Fund scheme which is transferred to local governments. This budget is used for all local public interests and the utilization is handed over to the local government, including stunting reduction. There is also a Special Allocation Fund and Co-Administration Fund which are transferred to institutions at the regions for the benefit of central affairs in the area of the district, also including the reduction in stunting. The local government itself has a budget that comes from regional revenues.

With these funds, districts carry out regional development programs and activities. Each year, the district government distributes its budget to all District Sector Agents (DSA) and sub-districts in accordance with the proposed budget plan. Moreover, the villages obtain a special budget from the central government through the Village Funds Allocation and from their district government.

District level stakeholders said that previously, the entire budget was fragmented between District Sector Agents (DSA). The handling of the stunting reduction program culminated in a budget that was not large enough to manage. However, stakeholders understood, accepted and adopted, and the budget increased meaningfully.

“Stunting handling budget for the 2020 plan, increased compared to the previous year of 2018 and 2019”.

(R4, district)

Stakeholders at the sub-district level state that they did not get a special budget from the DSA for the stunting reduction program. The sub-district only made activities and budget plans and proposed them to the district. The sub-district only functions as an implementer in these activities. Besides that, the sub-district must have the ability to lobby DSAs and DDPB at the district level to obtain a budget according to its needs.

“We at the sub-district level do not manage the District Revenue and Expenditure Budget (DREB) directly, we are only the executor of activities”.

(R99, sub-district)

“Yes, we have to be able to make good plans and be able to lobby DSA and DPPB to get the budget”.

(R82, sub-district)

Stakeholders at the village level said that they received a direct budget each year through Village Fund Allocation (VFA), which was used to finance village operational activities. The VFA, in accordance with central government regulations, can be used for nutrition-specific and -sensitive intervention activities. However, it was revealed that nutrition-specific activities only receive a budget allocation of around 0.3–4.4% of VFA, while nutrition-sensitive activities obtain an allocation of between 7.7–88.2%. This means that there are very small villages which allocate their activity budgets for nutrition-sensitive interventions. In contrast, there are villages that allocate very large budgets—almost 90% of VFA. However, all nutrition-specific and -sensitive intervention activities have not been integrated.

“Nearly 90% of our VFA is used to finance sensitive intervention activities, we admit that it has not been integrated with specific intervention”.

(R149, villages)

“Yes, I just allocated around 0.3% VFA for nutrition-specific intervention and have not been integrated with sensitive intervention”.

(R153, villages)

Convergence actions have not been realized in the budget at the village level. The implementation of convergence actions requires careful planning at the operational level, but the capacity of village planning has not been able to realize it. There are no activities facilitated by the central government, provinces, or districts/cities to address the village’s official planning capacity. Sub-districts, as an extension of the district’s hand in administration and coordination, should be able to help these villages. Thus, the solution carried out by the facilitator (academician) is to compile modules and guidelines for village-level stunting convergence planning, training, and mentoring.

### 3.7. Coverage

Stakeholders at the district, sub-district, and village levels affirm that the coverage of nutrition-specific interventions has been carried out by the health sector. However, there are two (2) indicators whereby the coverage has not been optimal—exclusive breastfeeding (35.5%) and the provision of iron tablets for teenage girls (26%).

“The main problem for nutrition-specific is exclusive breastfeeding that is still very low and also the provision of the iron tablet for teenage girls”.

(R11, district; R72, sub-district)

The coverage of nutrition-sensitive interventions such as ODF is still low (27.36%) in addition to proper sanitation (52%), clean water facilities (60.5%), and healthy houses (65%). In addition, seven villages faced food insecurity in the area. The main problems in the nutrition-sensitive sector in the district research site are the low ODF and lack of proper sanitation, especially in the management of waste problems. This determinant of health causes high cases of diarrhea and infectious diseases in toddlers and becomes one of the risk factors for stunting.

“ODF and garbage are the main problems in our district”.

(R8, district)

“ODF and litter are the cause of high diarrhea and infection in toddlers in our district”.

(R67, sub-district; R82, villages)

### 3.8. Sustainability

Stakeholders at the district level have implemented this convergence policy on an ongoing basis, but at the sub-district and village levels, they have not been able to do so. At the district level, there is already a regulation made by the Ministry of Home Affairs. On the contrary, there is no specific regulation that supports the sub-district and village levels. 

Anthropometric measurements conducted by the Integrated Health Post are ran by the Community Integrated Healthcare (CIH) cadres. District-level stakeholders have conducted an annual review of the measurement report. There are potential technical problems in uncontrolled anthropometric measurements. However, at the sub-district and village levels, they have not reviewed the results of anthropometric measurements. The review was only carried out by the PHC, but the results were not optimal, because the risk factors could not be analyzed properly. This is due to the weak coordination and capacity of sub-district and village stakeholders. This review of anthropometric measurements is very important because it is used in integrated planning activities for the following year.

“The results of anthropometric measurements carried out twice a year by cadres, it is not analyzed by sub-districts or villages. Only us, the Public Health Center, doing the analysis, but we are not optimal. What is the cause? Because there are no other inter-sectors, even though this interview is important for next year’s activities”.

(R83, sub-district)

## 4. Discussion

Our findings show that the convergence action strategy for accelerating stunting prevention and reduction can already be implemented at the district level, while the sub-district and village levels have not been implemented properly. This is due to the many obstacles that occur at the sub-district and village levels compared to districts. The barriers at the sub-district level are a matter of commitment, capacity, and weak coordination. Likewise, the barriers at the village level are issues of commitment, politics, capacity, and coordination.

At the sub-district level, all of them have formed the SRAT, but at the village level, only 50% has been achieved. Convergence action as a principal strategy in stunting reduction programs requires attention at the operational level. Leaders at the first line of the district, as agents and holders of political and bureaucratic authority in the district, must be able to ensure that the central policy is carried out up to the agency, which is the closest to the community.

As an agent, the district government will definitely accept the central order (principal), as long as there are legal and formal orders [26,27,28]. However, in regard to whether an accepted order is well adopted, implementing a national task requires a strong understanding of what material is ordered, and what a different policy has long been worked on with the existence of a new strategy in the form of convergence action. A complete understanding will result in wholehearted adoption and implementation.

The implementation of the convergence policy at the district level is due to encouragement from the Ministry of Home Affairs and the Provincial Government. They assess the convergence of stunting reduction management as a district government performance which is carried out every year; this is carried out so that it does not matter who the major is, as they will still have a high commitment to reducing stunting. However, for the sub-district and village governments, there are no regulations like in the district. Even at the village level, the political nuance is very strong. When the village chief and district leader are not in the same party, the village chief only supports them half-heartedly. In the other case, many nutrition-specific and -sensitive intervention activities have not worked as they had previously because of the impact of political leadership succession in the villages.

The findings of this study are different from the research in India, which found that coordination at higher levels is easier than at the village level; however, there are some similarities, such as that inter-level teamwork has different capacities as well as coordination [29]. The results of this study are supported by research by Pelletier et al., who state that the convergence of handling stunting is closely related to politics, the commitment of all stakeholders at all levels, and the reliable capacity of all stakeholders [30].

The results of our study indicate that a reduced implementation of the stunting reduction convergence at the sub-district level causes a heavier assignment for the PHC. Almost all nutrition-specific and -sensitive intervention activities became a burden to the PHC. The results of this study are the same as those in Odisha India, where the health sector has a strong dominance in the interventions that regard handling the stunting reduction program [17].

Nutrition-sensitive interventions should be carried out cross-sectoral at the sub-district level. On the other hand, specific interventions are only carried out by the PHC, whereas, at the sub-district level, there are many private PHCs. Their involvement is limited to only services that manage malnutrition, pregnancy check-ups, and immunizations. It may be best if the private PHC is involved in all nutrition-specific intervention services. However, the challenge is that the PPHC could not benefit from a government budget for handling the nutrition-specific problem.

A superficial understanding and a sub-district- and village-level capacity regarding the stunting intervention at the operational level affects the responsibility of handling stunting at the lowest level, which then returns to the health sector. In fact, the convergence action is aimed at determinants that are beyond the responsibility, ability, and authority of the health sector. The inter-sectoral approach at the operational level is constrained by feelings of a conflict of interest in sectoral implementers at the field level. The inter-sectoral mission and vision linkages need to be clearly described. For this reason, it requires the support from external local governments, whether from supra systems such as central and provincial governments, or outside parties such as academics. Academics can play a more neutral, balanced, but strong-enough role in the capacity of ‘agents’ in the field.

The implementation of convergence actions to accelerate stunting prevention and reduction has many barriers and challenges, such as the ineffectiveness of the program, inefficient allocation, and the utilization of resources [31]. Stunting prevention and reduction efforts will not succeed if carried out by individuals or by one sector only. The success of the stunting reduction can be completed faster if integration and convergence action efforts are carried out at the central, regional, and village levels [32]. The existence of clear responsibilities in resources, leadership, and governance between sectors in nutrition-specific and -sensitive interventions has a strong potential to support the acceleration of stunting reduction [33]. The developed strategy is carried out through collaborations and coordination across sectors at the central and regional levels, as well as through the provision of sustainable food, health, water, and nutrition [34]. Multisectoral action plans at various levels have been proven to reduce stunting [33,35,36].

Learning from other countries, almost all of them have taken a long time to see the impact of implemented convergence policies on stunting reduction, such as Peru, Thailand, Vietnam, Brazil, and PNG [37,38]. This also occurred in Afghanistan—at the beginning of the implementation of the convergence, there was no uniformity for nutrition-specific and -sensitive intervention activities in the community, although the government’s political commitment began to increase [39]. Another obstacle in implementing the convergence policy at the sub-district level is that they do not manage finances. Thus, the decision space for implementing the sub-district costs is narrow, while the districts and villages are wide [40].

The reluctance of the non-health sector in implementing convergence strategies suggests that stunting is absolutely seen as a health problem. The non-health sector perceives stunting as a downstream problem, not upstream, where sectors outside health are located. Practically speaking, convergence will be perceived as a burden to the budget of the non-health sector programs. This phenomenon is often faced in mainstreaming health in general.

The stunting reduction policy is the general mission and vision of the central government, with convergence actions as a strategy. On the other hand, sectors outside of health have their mission, vision, and priorities. There is a difference between the interests of the state and the main interests of the sectoral sector, which seems to cause a deflection in the achievement of goals. The difference in deflection complicates the level of stakeholder engagement. The same phenomenon occurs among programs in the health sector itself. Convergence in practice will have an impact on budget allocation and will create jealousy between health programs. 

The leaders are challenged with the ability to harmonize the national or principal’s mission line with the interest of these organizations (agents) and their programs. Managers need to be strengthened by the ability to identify and minimize deflection. In other words, the leader must be able to align the agent’s mission beyond the subject of convergence with the principal’s mission and vision.

Proper policy implementation is resulted from a holistic and comprehensive policy formulation. Policy makers need to ensure that implementation can be carried out at the operational level. Proper policy implementation must be supported by the ability to understand and the planning capacity of the implementers. The lesson from this case is that policy makers have not yet reached an understanding regarding what to do with the dissemination of policy content in the field. Furthermore, it requires the ability to implement the policies from both from a managerial and technical side. 

The key success of convergence implementation in this study is focused on the responsible agents and their roles: a strong leadership of the first-line district authority; increased funds for a technical conductor role by the DDPB; better advocating and agility of the District Health Agency; a strong coordination at the sub-district level; the commitment and consistency of the village leader. This result is in line with Avula’s opinion that all central, district, and community stakeholders work closely together, ensuring sustainable leadership and adequate funding [41].

Some of the limitations of this study include: the study did not calculate the prevalence of stunting, the results of this study cannot be generalized, and the observation time of the implementation runs one year. In addition, the mapping results for private PHC were obtained from the opinion of the head of the PHC and no triangulation was carried out on private PHC. The strength of this research is that the observation and document retrieval was carried out in all districts and villages, which focused on stunting in Cirebon District.

## 5. Conclusions

National policies in the convergence actions of stunting reduction have not been able to fully reach the lowest levels of government. Implementation of the policy tends to be positively acceptable, appropriate, and better in nutrition-specific intervention coverage at the sub-district and village levels. On the other hand, implementation is less carried out in terms of adoption, feasibility, fidelity, implementation cost, nutrition-sensitive intervention coverage, and sustainability aspects. Accelerating stunting reduction to achieve the SDG targets requires additional strategies at the operational level. The potential solutions proposed were: a stronger leadership of the first-line district authority, increased funds for a technical conductor by the Planning and Financial Board, better advocating and agility of the District Health Agency, a stronger coordination at the sub-district level, and a commitment and consistency of village leaders. In addition, the involvement of private PHC is very important to reduce the burden on PHC.

## Figures and Tables

**Table 1:** Implementation of Stunting Policy Mapping at The District Level.

Implementation Outcome of Stunting Policy	Baseline (BL)	Endline (EL)
Acceptability	Not all stakeholders at the district level accept the convergence policy for handling stunting because they do not understand stunting, the impact of stunting, and the importance of the convergence policy.	All district-level stakeholders accept the convergence policy for dealing with stunting. The Major and the District Secretary form a team to accelerate the reduction in stunting at the district level (SRAT).
Adoption	There have only been two out of eight actions in the convergence policy to accelerate stunting reduction carried out by stakeholders.	The district level has been able to adopt eight convergence actions. In the implementation of actions, three are led directly by the Major. District-level leaders are highly committed to accelerate stunting reduction. DDPB compiles an academic script and a draft of district regulations on the acceleration of stunting prevention, assisted by RWG. Local governments draft the village fund allocation regulations.
Appropriateness	All sectors are involved, but understanding is superficial.	All stakeholders say that convergence policies are right to deal with stunting. Nutrition-specific and -sensitive interventions are appropriately integrated into dealing with stunting. A better understanding of the budget execution for clear interventions.
Fidelity	District stakeholders have not been able to implement a convergence policy for dealing with stunting.	District stakeholders have been able to implement a convergence policy for dealing with stunting.
Implementation Cost	The budget for stunting programs is still fragmented in each sector agency, and the budget of the district is not large enough.	DDPB becomes the coordinator in making integrated planning. The stunting budget has been integrated and there is an increase from DREB.
Feasibility	The district- and village-level governments have not been able to plan integrated nutrition-specific and -sensitive intervention activities.	The sub-district- and village-level governments have been able to plan for stunting reduction through integrated nutrition-specific and -sensitive programs. All DSAs can integrate programs and budgets well. The private sector and academia are starting to play a role in assisting the district-level government in nutrition-specific and -sensitive programs.
Coverage	The scope of nutrition-specific interventions is broad and equitable; they are of poor quality. Limited range of sensitive interventions.	Broad, equitable coverage of nutrition-specific interventions with better quality. Better coverage of sensitive interventions.
Sustainability	The results of measuring the nutritional status of children under five through anthropometry conducted by the health sector have not yet been published and reviewed. Inter-sectoral coordination has not gone well.	The results of the measurement of nutritional status reported by the District Health Office/ Agency (DHO/A) are published to all stakeholders. In addition, a joint review of the problems and strategies for solving them is also carried out. Inter-sectoral coordination has been running quite harmoniously. The results of anthropometric reviews are used in planning the nutrition-specific and -sensitive interventions for the coming year.

**Table 2:** Implementation of Stunting Policy Mapping at the Sub-District Level.

Implementation of Stunting Policy	Sub-District A		Sub-District B		Sub-District C		Sub-District D		Sub-District E		Sub-District F	
	BL	EL	BL	EL	BL	EL	BL	EL	BL	EL	BL	EL
Acceptability	−	+	−	+	−	+	−	+	−	+	−	+
Adoption	−	−	−	−	−	−	−	−	−	−	−	−
Appropriateness	−	+	−	+	−	+	−	+	−	+	−	+
Feasibility	−	−	−	−	−	−	−	−	−	−	−	−
Fidelity	−	+	−	−	−	−	−	−	−	−	−	−
Implementation Cost	−	+	−	−	−	−	−	−	−	−	−	−
Coverage-Specific Intervention	+	+	+	+	+	+	+	+	+	+	+	+
Coverage-Sensitive Intervention	−	−	−	−	−	−	−	−	−	−	−	−
Sustainability	−	+	−	−	−	−	−	−	−	−	−	−

Note: Acceptability: Understand and accept the convergence policy and form teamwork to handle stunting. Adoption: Adopt an integrated nutrition-specific and -sensitive intervention policy and create supporting regulations. Appropriateness: Relevance, the usefulness of nutrition-specific and -sensitive interventions in dealing with stunting. Feasibility: Nutrition-specific and -sensitive intervention policies are feasible. Fidelity: Able to carry out the intervention because it has made an integrated nutrition-specific and -sensitive intervention plan. Implementation Cost: Allocating the VFA budget for integrated nutrition-specific and sensitive intervention activities. Coverage: Target the number of people within the first 1000 days of birth receiving nutrition-specific and -sensitive intervention benefits. Sustainability: Coordinate reviews of nutritional status measurements and create integrated solutions on an ongoing basis. −: not carried out yet; +: already carried out.

**Table 3:** Implementation of Stunting Policy Mapping at the Village Level (Endline).

Implementation of Stunting Policy	Village (Vill) A	Vill B	Vill C	Vill D	Vill E	Vill F	Vill G	Vill H	Vill I	Vill J
Acceptability	−	−	+	−	+	−	+	+	−	+
Adoption	−	−	−	−	−	−	−	−	−	−
Appropriateness	−	+	−	+	−	+	−	+	−	+
Feasibility	−	−	−	−	−	−	−	−	−	−
Fidelity	−	−	−	−	−	−	−	−	−	−
Implementation Cost	−	−	−	−	−	−	−	−	−	−
Coverage-Specific Intervention	+	+	+	+	+	+	+	+	+	+
Coverage-Sensitive Intervention	−	−	−	−	−	−	−	−	−	−
Sustainability	−	−	−	−	−	−	−	−	−	−

Note: Acceptability: Understand and accept the convergence policy and form teamwork to handle stunting. Adoption: Adopt an integrated nutrition-specific and -sensitive intervention policy and create supporting regulations. Appropriateness: Relevance, the usefulness of nutrition-specific and -sensitive interventions in dealing with stunting. Feasibility: Nutrition-specific and -sensitive intervention policies are feasible. Fidelity: Able to carry out the intervention because it has made an integrated nutrition-specific and -sensitive intervention plan. Implementation cost: Allocating the VFA budget for integrated nutrition-specific and -sensitive intervention activities. Coverage: Target the number of people within the first 1000 days of birth receiving nutrition-specific and -sensitive intervention benefits. Sustainability: Coordinate reviews of nutritional status measurements and create integrated solutions on an ongoing basis. −: not carried out yet; +: already carried out.

**Table 4:** Implementation of Stunting Policy Mapping in Primary Care.

Implementation of Stunting Policy	Public Health Center (PHC)	Private Primary Health Care (PPHC)
Acceptability	The nutrition-specific intervention program has been well understood by the Public Health Center because it is a program that achieves the minimum service standards of the PHC.	Nutrition-specific intervention programs are well understood by private primary services but are not used by them.
Adoption	The Public Health Center has carried out nutrition-specific interventions including the provision of iron and folic acid supplementation (IFAS); additional nutrition interventions for pregnant women and toddlers with calory and energy malnutrition; exclusive breastfeeding counseling and promotion; baby and toddler feeding counseling; management of malnutrition; growth monitoring; calcium, Vitamin A, and zinc supplementation; pregnancy checks, immunizations, administration of deworming and IMCI.	The PPHC only runs a part of nutrition-specific intervention programs, such as the management of malnutrition, antenatal care, and immunizations.
Appropriateness	The PHC carries out all nutrition-specific and some -sensitive interventions.	The PPHC can only perform certain nutrition-specific interventions and is limited to patients who come for treatment.
Feasibility	Nutrition-specific interventions are very feasible to be carried out by the PHC.	It should be very feasible for nutrition-specific interventions to be carried out by the PPHC.
Fidelity	The PHC is able to carry out nutrition-specific interventions because it has more resources than the PPHC.	The PPHC is able to carry out nutrition-specific interventions, but resources are limited, so only a few nutrition-specific interventions such as antenatal care, immunizations, IFAS administration, and poor nutrition management are carried out.
Implementation Cost	The PHC manage the Special Operational Fund (SOF) budget from the Ministry of Health which can be used to finance nutrition-specific intervention activities. For additional nutrition interventions, IFAS, facilities, and infrastructure were dropped from the Ministry of Health and the District and Provincial Health Offices.	The PPHC did not receive any budget from central and local governments for nutrition-specific interventions.
Coverage	Covers all targets regarding stunting sufferers and families.	Very limited.
Sustainability	The nutrition-specific intervention program is a program that is carried out continuously by the PHC.	The nutrition-specific intervention program is not a program in the PPHC. The coordination of the PHC officers is going well but is not working yet in private primary healthcare.

## Data Availability

Not applicable.

## References

[1] UNICEF, WHO, World Bank Group (2021). Joint Malnutrition Estimates Levels and Trends.

[2] de Onis M., Branca F. (2016). Childhood stunting: A global perspective. Matern. Child. Nutr..

[3] Woldehanna T., Behrman J.R., Araya M.W. (2017). The effect of early childhood stunting on children’s cognitive achievements: Evidence from young lives Ethiopia. Ethiop. J. Health Dev..

[4] Adair L.S., Fall C.H.D., Osmond C., Stein A.D., Martorell R., Ranirez-Zea M., Sachdev H.S., Dahly S.L., Bas I., Norris S.A. (2013). Associations of linear growth and relative weight gain during early life with adult health and human capital in countries of low and middle income: Findings from five birth cohort studies. Lancet.

[5] Victora C.G., Adair L., Fall C., Hallal P.C., Martorell R., Richter L., Sachdev H.S., for the Maternal and Child Undernutrition Study Group (2008). Maternal and child undernutrition: Consequences for adult health and human capital. Lancet.

[6] (2019). Scaling Up Nutrition. The SUN Movement Strategy and Roadmap. https://scalingupnutrition.org/about-sun/the-sun-movement-strategy/.

[7] Kementerian PPN/Bappenas (2018). Pedoman Pelaksanaan Intervensi Penurunan Stunting Terintegrasi di Kabupaten/Kota.

[8] Tim Nasional Percepatan Penanggulangan Kemiskinan (2018). Panduan Konvergensi Program/Kegiatan Percepatan Pencegahan Stunting.

[9] Balarajan Y., Levinson F.J. (2013). Addressing Malnutrition Multisectoral. What Have We Learned from Recent International Experiences?.

[10] Huicho L., Vidal-Cárdenas E., Akseer N., Brar S., Conway K., Islam M., Juarez E., Rappaport A.I., Tasic H., Vaivada T. (2020). Drivers of stunting reduction in Peru: A country case study. Am. J. Clin. Nutr..

[11] Brar S., Akseer N., Sall M., Conway K., Diouf I., Everett K., Islam M., Sylmang Sène P.I., Tasic H., Wigle J. (2020). Drivers of stunting reduction in Senegal: A country case study. Am. J. Clin. Nutr..

[12] Conway K., Akseer N., Subedi R.K., Brar S., Bhattarai B., Dhungana R.R., Islam M., Mainali A., Pradhan N., Tasic H. (2020). Drivers of stunting reduction in Nepal: A country case study. Am. J. Clin. Nutr..

[13] Hossain M., Choudhury N., Abdullah K.A.B., Mondal P., Jackson A.A., Walson J., Ahmed T. (2017). Evidence-based approaches to childhood stunting in low and middle-income countries: A systematic review. Arch. Dis. Child..

[14] de Onis M., Dewey K.G., Borghi E., Onyango A.W., Blossner M., Daelmans B., Piwoz E., Branca F. (2013). The World Health Organization’s global target for reducing childhood stunting by 2025: Rationale and proposed actions. Matern. Child. Nutr..

[15] Gani A.A., Hadju V., Syahruddin A.N., Otulawa A.S., Palutturi S., Thaha A.R. (2021). The effect of convergent action on reducing stunting prevalence in under-five children in Banggai District, Central Sulawesi, Indonesia. Gac. Saint..

[16] Remans R., Pronyk P.M., Fanzo J.C., Chen J., Palm C.A., Nemser B., Muniz M., Radunsky A., Hadera A., Coulibaly M. (2011). Multisector intervention to accelerate reductions in child stunting: An observational study from 9 sub-Saharan African countries. Am. J. Clin. Nutr..

[17] Kim S.S., Avula R., Ved R., Kohli N., Singh K., van den Bold M., Kadiyala S., Menon P. (2017). Understanding the role of intersectoral convergence in the delivery of essential maternal and child nutrition interventions in Odisha, India: A qualitative study. BMC Public Health.

[18] Kementerian Kesehatan, Badan Pusat Statistik (2019). Laporan Pelaksanaan Intergrasi Susenas Maret 2019 dan SSGBI Tahun 2019.

[19] Peters D.H., Adam T., Alonge O., Agyepong I.A., Tran N. (2013). Implementation research: What it is and how to do it. BMJ.

[20] Metz A., Jensen T., Farley A., Boaz A. (2022). Is implementation research out of step with implementation practice? Pathways to effective implementation support over the last decade. Implement. Res. Pract..

[21] Spencer R., Ptyce J.M., Walsh J., Nathan P.E. (2014). Philosophical Approaches to Qualitative Research. Oxford Handbook of Qualitative Research.

[22] Lindsay P., Nathan P.E. (2014). Content Analysis. Oxford Handbook of Qualitative Research.

[23] Saldana J., Nathan P.E. (2014). Coding and Analysis Strategis. Oxford Handbook of Qualitative Research.

[24] Palmberger M., Ginrich A., Flick U. (2014). Qualitative Comparative Practice: Dimension, Cases and Strategy. The Sage Handbook of Qualitative Data Analysis.

[25] Cresswell J.W. (2014). Research Design. Qualittaive, Quantitative and Mixed Methods Approaches.

[26] Nguyen H. (2011). The principle-agent problems in health care: Evidence from prescribing patterns of private providers in Vietnam. Health Policy Plan..

[27] Brinkerhoff D.W., Bossert T. (2014). Health governance: Principle-agent linkages and health system strengthening. Health Pol. Plan..

[28] Song H., Yu S., Sun T. (2020). Reducing the quality risk of elderly care services in government procurement from market-oriented private providers through *ex-ante* policy design: Lessons from the principle-agent theory analysis. BMC Health Serv. Res..

[29] Rajpal S., Joe W., Kim R., Kumar A., Subramanian S.V. (2020). Child undernutrition and convergence of multisectoral interventions in India: An econometric analysis of National Family Health Survey 2015–2016. Front. Public. Health.

[30] Pelletier D.L., Frongillo E.A., Gervais S. (2012). Nutrition agenda setting, policy formulation and implementation: Lessons from the Mainstreaming Nutrition Initiative. Health Policy Plan..

[31] Hartotok H., Absori A., Dimyati K., Santoso H. (2021). Stunting prevention policy as a form of child health rights legal protection. Maced. J. Med. Sci..

[32] Black R.E., Allen L.H., Bhutta Z.A., Caulfield L.e., de Onis M., Ezzati M., Mathers C., Rivera J., Maternal and Child Undernutrition Study Group (2008). Maternal and child undernutrition: Global and regional exposures and health consequences. Lancet.

[33] Bhuta Z.A., Akseer N., Keats E.C., Vaivada T., Baker S., Horton S.E., Katz J., Menon P., Piwoz E., Shekar M. (2020). How countries can reduce child stunting at scale: Lessons from exemplar countries. Am. J. Clin. Nutr..

[34] Laillou A., Gauthier L., Wieringa F., Berger J., Chea S., Poirot E. (2020). Reducing malnutrition in Cambodia. A modeling exercise to prioritize multisector interventions. Matern. Child. Nutr..

[35] Rasanathan K., Damji N., Atsbeha T., Drisse M.-N.B., Davis A., Dora C., Karam A., Kuruvilla S., Mahon J., Neira M. (2015). Ensuring multisectoral action on the determinants of reproductive, maternal, newborn, child, and adolescent health in the post-2015 era. BMJ.

[36] Gillespie S., Haddad L., Mannar V., Menon P., Nisbett N. (2013). Maternal and Child Nutrition Study Group. The politics of reducing malnutrition: Building commitment and accelerating progress. Lancet.

[37] National Planning Commission (2012). Multi-Sector Nutrition Plan, for Accelerating the Reduction of Maternal and Child under-Nutrition in Nepal 2013–2017 (2023).

[38] Kathuria A.K., Arur A., Digwaleu-Kariko E.A.B. (2019). Success Stories with Reducing Stunting: Lessons for PNG.

[39] Kim C., Mansoor F., Paya P.M., Ludin M.H., Ahrar M.J., Mashal M.O., Todd C.S. (2020). Multisector nutrition gains amids evidence scarcity: Scoping review of policies, data and interventions to reduce child stunting in Afghanistan. Health Res. Pol. Syst..

[40] Bossert T.J. (2016). Decision space and capacities in the decentralization of health services in Fiji. Int. J. Health Policy Manag..

[41] Avula R., Nguyen P.H., Tran L.M., Kaur S., Bhatia N., Sarwal R., de Wagt A., Chaudhery D.N., Menon P. (2022). Reducing childhood stunting in India: Insights from four subnational success cases. Food Secur..

